# Possible value of antifibrotic drugs in patients with progressive fibrosing non-IPF interstitial lung diseases

**DOI:** 10.1186/s12890-019-0937-0

**Published:** 2019-11-12

**Authors:** Sebastiano Emanuele Torrisi, Nicolas Kahn, Julia Wälscher, Nilab Sarmand, Markus Polke, Kehler Lars, Monika Eichinger, Claus Peter Heussel, Stefano Palmucci, Francesca Maria Sambataro, Gianluca Sambataro, Domenico Sambataro, Carlo Vancheri, Michael Kreuter

**Affiliations:** 1 Center for interstitial and rare lung diseases, Pneumology, Thoraxklinik, University of Heidelberg, Germany and German Center for Lung Research, Heidelberg, Germany; 2 Department of Clinical and Experimental Medicine, Regional Referral Centre for Rare Lung Diseases, A.O.U. Policlinico-Vittorio Emanuele, University of Catania, Catania, Italy; 3 Department of Diagnostic and Interventional Radiology with Nuclear Medicine, Thoraxklinik, University of Heidelberg and Translational Lung Research Center Heidelberg, member of the German Center for Lung Research, Heidelberg, Germany; 4 Radiology I Unit, Department of Medical Surgical Sciences and Advanced Technologies, University Hospital “Policlinico-Vittorio Emanuele”, Catania, Italy; 5 Department of Imaging, Azienda Ospedaliero Universitaria Città della Salute e della Scienza, CTO Hospital, Via Zuretti 29, 10126 Turin, Italy; 6 Artroreuma s.r.l. Outpatient of Rheumatology accredited with the National Health System, c.so S. Vito 53, 95030 Mascalucia, CT Italy; 7 Department of Clinical and Experimental Medicine, Internal Medicine Unit, Cannizzaro Hospital, University of Catania, Via Messina 829, 95100 Catania, Italy

**Keywords:** Interstitial lung disease, Nintedanib, Pirfenidone, Progressive fibrosing interstitial lung diseases, Real-world experience, IPAF

## Abstract

**Background:**

Fibrosing, non-idiopathic pulmonary fibrosis (non-IPF) interstitial lung diseases (fILDs) are a heterogeneous group of diseases characterized by a different amount of inflammation and fibrosis. Therapy is currently based on corticosteroids and/or immunomodulators. However, response to these therapies is highly variable, sometimes without meaningful improvement, especially in more fibrosing forms. Pirfenidone and nintedanib have recently demonstrated to reduce functional decline in patients with IPF. However, their antifibrotic mechanism makes these two drugs an interesting approach for treatment of fibrosing ILDs other than IPF.

**Objectives:**

We here report our experience with antifibrotic drugs in fibrosing non-IPF ILDs patients having a progressive phenotype during immunosuppressive therapy.

**Methods:**

Patients with a multidisciplinary team diagnosis of fibrosing non-IPF ILDs experiencing a progressive phenotype during treatment with corticosteroids and/or immunomodulators between October-2014 and January-2018 at our tertiary referral Center for ILDs were retrospectively analyzed. Antifibrotic therapy was administered after application with the respective health insurance company and after consent by the patient. Pulmonary-function-tests and follow-up visits were performed every 6 ± 1 months.

**Results:**

Eleven patients were treated with antifibrotic drugs (8 males, mean age 62 ± 12.8 years, mean FVC% 62.8 ± 22.3, mean DLCO% 35.5 ± 10.7, median follow-up under antifibrotic treatment 11.1 months). Patients had a diagnosis of unclassifiable ILD in 6 cases, pleuroparenchymal fibroelastosis in 2 cases, idiopathic-NSIP in 1 case, asbestos-related ILD in 1 case and Hermansky-Pudlak syndrome in 1 case. Treatment before antifibrotics consisted of corticosteroids in all patients: 5 combined with Azathioprin, 1 with either methotrexate or cyclophosphamide (i.v.). Ten patients were treated with pirfenidone (2403 mg/die) and 1 with nintedanib (300 mg/die). Median FVC was 56, 56, 50%, at time points − 24, − 12, − 6 before initiation, 44% at time of initiation and 46.5% at 6 months after initiation of antifibrotic treatment. Antifibrotic treatment was generally well tolerated with a need of dose reduction in 2 cases (rash and nausea) and early termination in 3 cases.

**Conclusions:**

Antifibrotic treatment may be a valuable treatment option in patients with progressive fibrosing non-IPF ILD if currently no other treatment options exist. However, prospective, randomized clinical trials are urgently needed to assess the real impact of antifibrotic therapy in these patients.

## Background

Interstitial lung diseases comprise a heterogeneous group of almost 200 entities characterized by a different amount of inflammation and/or fibrosis [1, 2]. Idiopathic Pulmonary Fibrosis (IPF) is the most frequent and aggressive form, representing the prototype of progressive fibrosing interstitial lung diseases [3, 4]. However, recent evidence has shown that also other non-IPF fibrosing interstitial lung diseases may, similarly to IPF, reveal a progressive phenotype (PF-ILDs) characterized by a rapid functional decline, worsening of symptoms and a detrimental prognosis [5–9]. According to a recent study by Olson et al., the prevalence of this subset of patients has been estimated at 0.22–2 per 10,000 persons in Europe and 2.8 per 10,000 persons in USA, thus representing a considerable number [10].

Therapy of PF-ILDs is currently based on corticosteroids and/or immunomodulators. However, response to these therapies is highly variable, sometimes without meaningful improvement [11]. In recent years two antifibrotic drugs, pirfenidone and nintedanib, have been developed and approved for the treatment of IPF [12–17]. Their impact on the course of other fibrosing ILDs is unknown. However, given some pathobiological and clinical similarities between PF-ILDs and IPF, both pirfenidone and nintedanib, may represent an interesting and reasonable approach also for PF-ILDs [18–21].

We here report our experience with antifibrotic drugs in fibrosing non-IPF ILDs patients with a progressive phenotype despite immunosuppressive therapy.

## Methods

### Study population

A retrospective analysis of the database of our tertiary referral Center identified all patients with a multidisciplinary team (MDT) diagnosis of fibrosing non-IPF ILDs that experienced a progressive decline in lung function during treatment with corticosteroids and/or immunomodulators between October-2014 and January-2018. At the time of diagnosis, a complete evaluation of medical history, serological data including autoantibodies, all comorbidities and related treatments were assessed. A rheumatologic evaluation was also required to interpret any rheumatologic sign and serological data [1, 22–24]. Each patient underwent high resolution computed tomography (HRCT) exams at baseline time and on follow-up every 6–12 months. In-house software YACTA was used to automatically quantify lung density histogram. Longitudinal changes in the 40th and 80th percentiles of attenuation histogram were assessed [25]. If possible, Bronchoalveolar lavage (BAL) and transbronchial cryobiopsy were also performed. Age, physiology and distinct comorbidities were used to calculate the TORVAN index, an index predictive of mortality recently validated for IPF [26]. Patients were considered to have a progressive phenotype if there was evidence of any of the following criteria: a relative decline of ⩾10% in forced vital capacity (FVC); a relative decline of ⩾15% in diffusing capacity of the lung for carbon monoxide (DLCO); or worsening symptoms or a worsening radiological appearance accompanied by a ⩾5–< 10% relative decrease in FVC within a 24-month period prior to antifibrotic therapy initiation [5, 6]. Patients underwent routinely follow-up visits and pulmonary function tests (FVC and DLCO) every 6 ± 1 months. Antifibrotic therapy, either pirfenidone or nintedanib, was introduced after a proven progression of the disease and lack of response to corticosteroids and/or immunomodulators, after approval of the respective health insurance company and according to German laws for off-label use and after consent by the patient. As in IPF patients, pirfenidone was given as continuous oral treatment at a dose of 2403 mg·day^− 1^ (3 capsules three times·day^− 1^) and nintedanib 300 mg·day^− 1^ (1 capsule twice·day^− 1^) [12–17]. Patients under antifibrotic therapy were reevaluated every 6–12 weeks after initiation according to the standard of care in our department. Liver function monitoring was conducted on a monthly basis. All adverse events (related and not related to treatment), treatment compliance and interruptions for any reason were also recorded at each treatment visit. To assess difference in one-year mortality between PF-ILD and IPF, a comparison with a cohort of 257 IPF patients collected in our center was also performed. All clinical information was obtained from medical records. Pulmonary function tests (FVC and DLCO) were performed according to the ATS/ERS guidelines.

### Statistical analysis

Characteristics of the study population were expressed as median (interquartile range) or as percentage of the relative frequency as appropriated. Wilcoxon test for paired data was used to assess differences in median FVC between each follow-up time. Kaplan-Meier survival analysis was used to assess overall survival. All the statistical analyses were performed using STATA/IC 14.2 version. A *p* value less than 0.05 was considered significant.

## Results

**Fig. 1:**
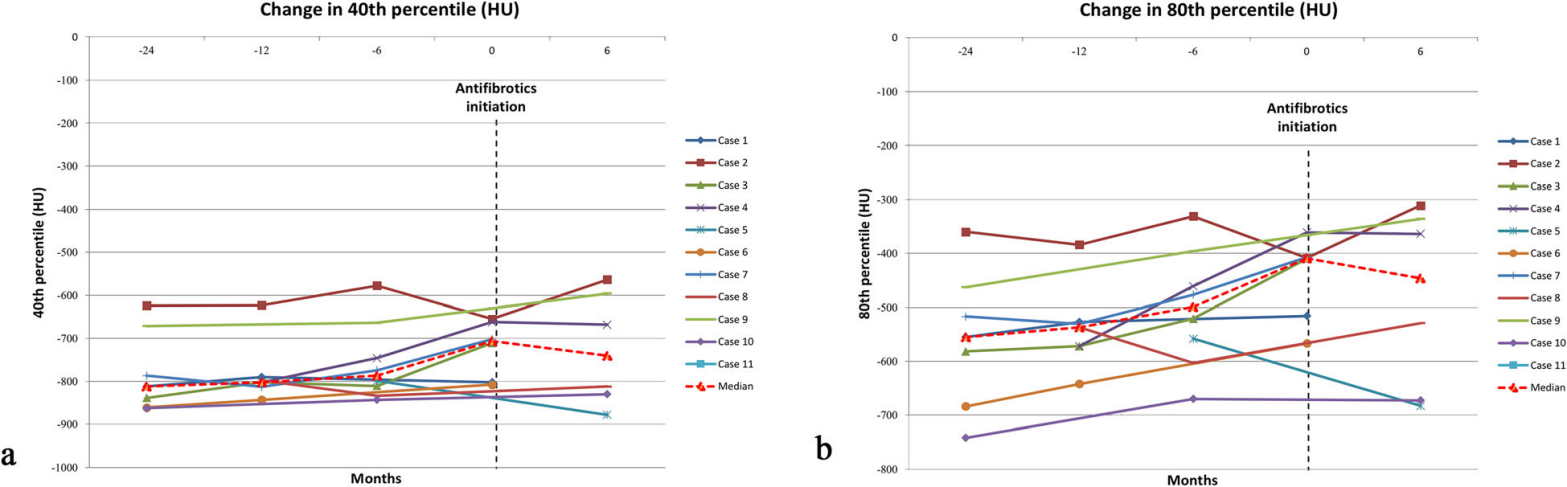
Change in the percentiles (Hounsfield units) of attenuation histogram over the time. Panel **a** demonstrates change in the 40^th^ percentiles while panel **b** demonstrates change in the 80^th^ percentiles

Eleven patients were included in the analysis. Eight were males (72.72%) and 3 were females (27.27%). There were 5 former smokers (45.45%) and 6 never-smokers (54.54%). The mean age was 62.09 ± 12.80 and 63.72 ± 12.72 years at diagnosis and at antifibrotic initiation respectively. The mean FVC % predicted was 62.82 ± 22.30 while the mean DLCO % predicted was 35.55 ± 10.74 before initiation of the antifibrotic therapy. The mean TORVAN index was 17.18 ± 5.13. Patients were followed for a median follow-up time of 16.6 months before and 11.1 months under antifibrotic treatment. Visual assessment of HRCT findings at baseline is showed in Table 1 while Fig. 1 reports automatic histogram-based assessment of 40th and 80th percentiles over the time. According to this result, a significant increase of both indices was observed before antifibrotic initiation followed by a stabilization. Bronchial alveolar lavage was performed in 8 cases before immunosuppressive therapy demonstrating a predominant neutrophilia (12%) in 4 cases, a notable eosinophilia (10%) in one case and no significant lymphocytosis.

**Table 1:** Baseline characteristics of patients

	Mean ± SD or n (%)	Median (range interquartile)
Age at diagnosis (years)	62.09 ± 12.80	63 (50, 76)
Age at initiation of antifibrotic therapy	63.72 ± 12.72	
Male	8 (72.72)	
Former smoker	5 (45.45)	
Never smoker	6 (54.54)	
Cryobiopsy	9 (81.81)	
Follow-up time (days) since diagnosis		903 (381, 1489)
Follow-up time (days) since antifibrotic initiation		333 (156, 421)
FVC% pred at baseline	62.82 ± 22.30	52.7 (49, 77)
DLCO% pred at baseline	35.55 ± 10.74	34 (29, 37.1)
TORVAN index (points)	17.18 ± 5.13	19 (13, 21)
HRCT features		
Reticulations	7 (63.6)	
Traction bronchiectasis	8 (72.7)	
Honeycombing	2 (27.2)	
Ground-glass	7 (63.6)	
Consolidations	3 (27.2)	
Mosaic attenuation	2 (27.2)	
Upper-mid lung predominance	3 (27.2)	
Lower lung predominance	4 (36.3)	
Prednisone	11 (100)	
Daily dosage (mg)	16.81 ± 11.18	
Azathioprin	5 (45.04)	
Daily dosage (mg)	150	
Methotrexate	1 (9,09)	
Weekly dosage (mg)	7.5	
Cyclophosphamide	1 (9.09)	
Dosage i.v. per cycle (mg)	1000

Transbronchial cryobiopsy was performed in 9 patients (81.8%). After a multidisciplinary team discussion, patients were classified as unclassifiable-ILD in 6 cases, pleuroparenchymal fibroelastosis (PPFE) in 2 cases, idiopathic-NSIP in 1 case, asbestos-related ILD in 1 case and Hermansky-Pudlak syndrome in 1 case. Of these, three patients may be also considered as interstitial pneumonia with autoimmune features (IPAF) according to established criteria (Table 2) [20]. Treatment before antifibrotic drug initiation consisted of corticosteroids (prednisone mean dosage 16.8 ± 11.1 mg/die) in all patients: 5 combined with Azathioprin (150 mg/die), 1 with either methotrexate (7,5 mg/week) or cyclophosphamide (1000 mg i.v. per cycle) (Table 1). After a proven clinical and functional progression, ten patients were treated with pirfenidone (2403 mg/die) and 1 with nintedanib (300 mg/die). Of the 11 patients, 6 continued prednisone (5 mg/die) for a mean time of 8.5 months and 2 continued Azathioprin for a mean time of 4 months after antifibrotic initiation. Median time of antifibrotic treatment was 11.1 (5.2, 14) months.

**Table 2:** Detailed characteristics of patients

Characteristics	Case 1	Case 2	Case 3	Case 4	Case 5	Case 6	Case 7	Case 8	Case 9	Case 10	Case 11
BMI	24	37	23	19	30	23	21	31	25	21	31
Smoking history (pack years)	Former (12 py)	Former (70 py)	Never	Never	Never	Never	Former (20 py)	Former (15 py)	Former (25 py)	Never	Never
ILD relevant exposure	no	no	no	Powders, detergents	no	no	no	Cement powders	powders	no	no
Autoantibodies	ANA 1:80; ENA negative	ANA 1:320; ENA negative	ANA 1:640; ENA negative	negative	ANA 1:80; ENA negative	ANA 1:1280; ENA negative	ANA 1:160; ANCA 1:32; ENA negative	ANA 1:320; ENA negative	ANA 1:320; ENA negative	ANA 1:160; ENA negative	ANA 1:160; ENA negative
FVC L (%) at baseline	1.9 (63)	1.82 (48)	2.74 (77)	1.26 (52.7)	2.1 (49)	2.31 (52.7)	4.28 (101)	3.37 (97.7)	3 (73)	0.93 (26.5)	1.47 (51.5)
DLCO % at baseline	38	35	unable	27.4	27	30	64	29	37.1	33	35
TORVAN index	19	21	19	22	14	24	13	17	7	12	21
BAL findings at initial diagnosis	Macrophages 65%, Lymphocytes 13%, Neutrophils 22%	Macrophages 85%, Lymphocytes 12%, Neutrophils 3%, Eosinophils 0%	Macrophage 76%, Lymphocytes 10%, Neutrophils 9%, Eosinophils 3%, Mastcells 2%	/	Macrophage 89%, Lymphocytes 9%, Eosinophils1%	Macrophages 89%, Lymphocytes 14%, Neutrophils 2%	Macrophage 96%, Lymphocytes 2%, Neutrophils 2%	Macrophage 91%, Lymphocytes 9%, Neutrophils 10%	Macrophages 77%, Lymphocytes 5%, Neutrophils 8%, Eosinophils 10%	/	/
Histological pattern	NSIP	/	Indeterminate	Compatible with PPFE	Indeterminate	Indeterminate	Indeterminate	UIP-like	Indeterminate	/	Indeterminate
LTOT at initiation of antifibrotic therapy	Yes	Yes	No	No	No	No	No	Yes	Yes	No	Yes
Distance at 6MWT (meters)	425	/	578	363	313	402	540	323	480	418	/
MDT diagnosis	iNSIP	uILD (IPAF)	uILD (IPAF)	PPFE	uILD	uILD (IPAF)	uILD	Asbestos-associated fibrosis	ILD in Hermansky-Pudlak syndrome	PPFE	uILD
Treatment before antifibrotics	Pred. 30 mg; AZT 125 mg; NAC 1800 mg	Pred. 15 mg; AZT 150 mg; MTX 7,5 mg	Pred. 30 mg; AZT 150 mg	Pred. 10 mg;	Pred. 10 mg;	Pred. 7,5 mg; AZT 150 mg; NAC 1800 mg	Pred. 7,5 mg; Cycl. (6 cycles)	Pred. 15 mg; AZT 150 mg	Pred. 10 mg;	Pred. 10 mg;	Pred. 40 mg;
Antifibrotic treatment	Pirf 2403 mg	Nint. 300 mg	Pirf 2403 mg	Pirf 1602 mg	Pirf 2403 mg	Pirf 2403 mg	Pirf 2403 mg	Pirf 2403 mg	Pirf 1602 mg	Pirf 2403 mg	Pirf 2403 mg

Footnotes: *BMI* Body mass index, *FVC* Forced Vital capacity, *DLCO* Diffusing Capacity for carbon monoxide, *BAL* Bronchial alveolar lavage, *LTOT* long term oxygen therapy, *MDT* multidisciplinary discussion, *iNSIP* idiopathic-Non specific interstitial Pneumonia, *uILD* unclassifiable Interstitial lung disease, *PPFE* pleuroparenchymal fibroelastosis, *Pred.* prednisone, *AZT* Azathioprin, *MTX* Methotrexate, *Cycl.* cyclophosphamide, *Pirf.* Pirfenidone, *Nint.* Nintedanib

**Fig. 2:**
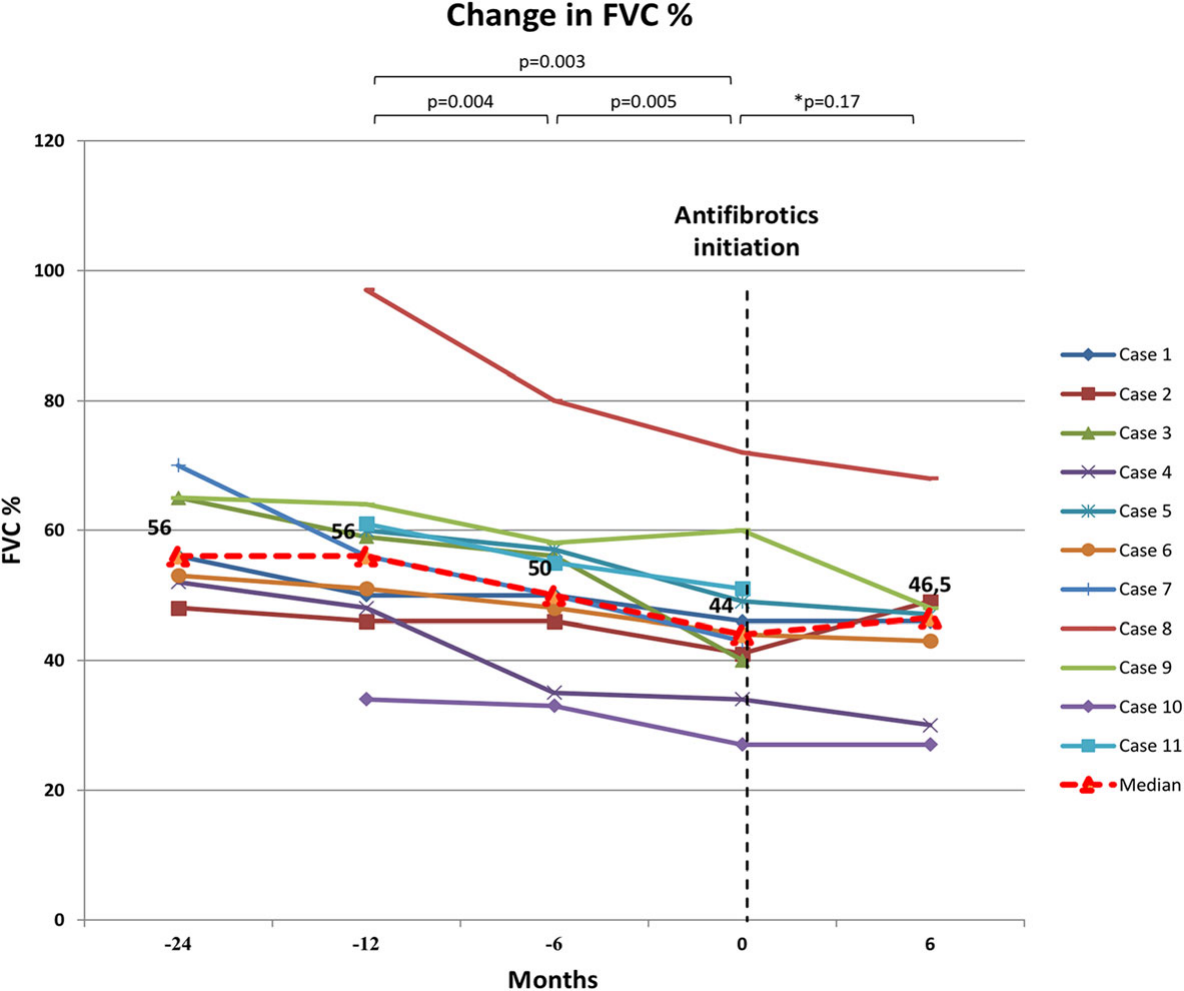
Change in FVC % predicted over the time. Footnotes: *this median difference was calculated comparing data of only eight patients as three patients died before the 6 months of follow-up since antifibrotics initiation

**Fig. 3:**
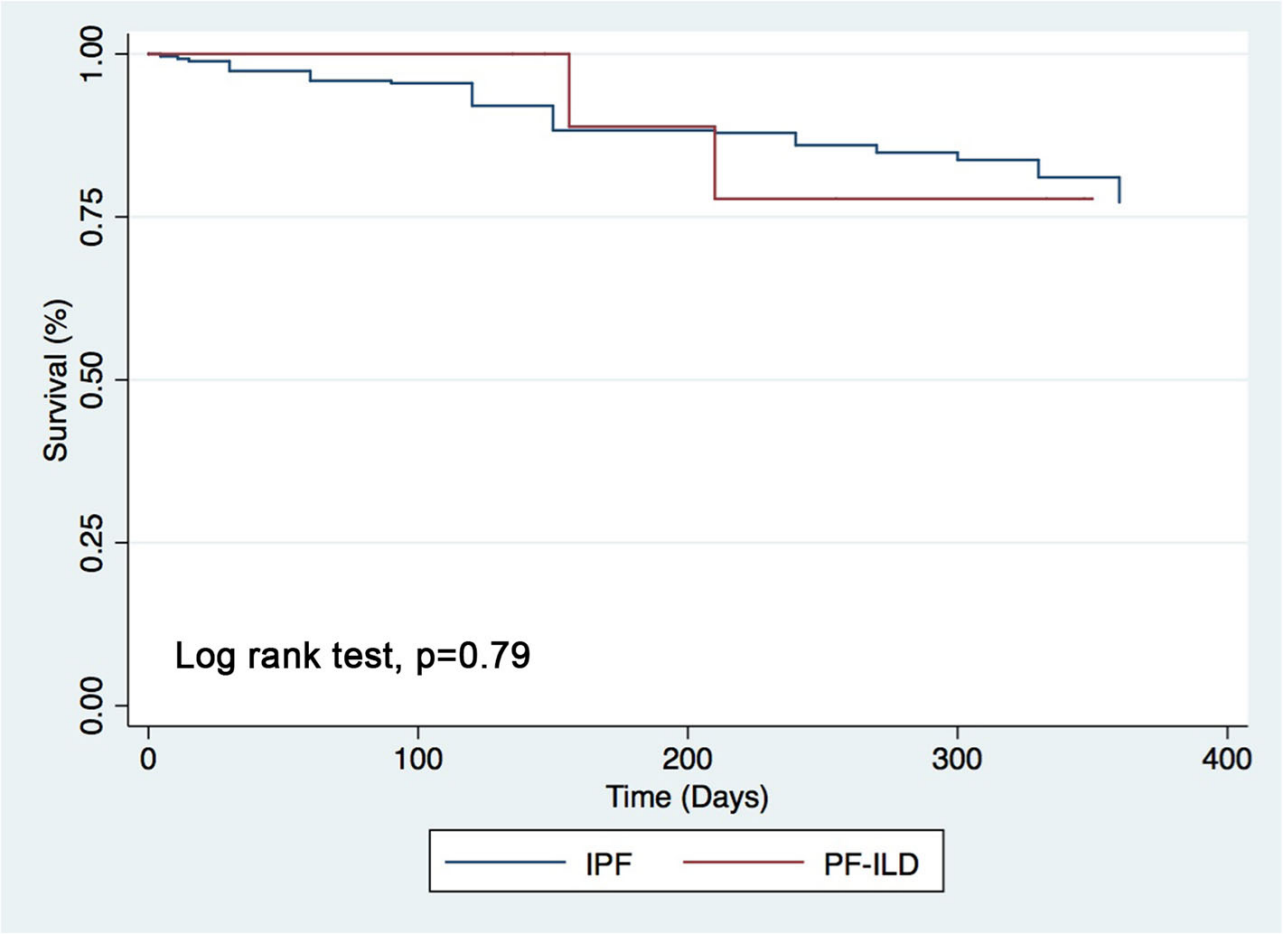
One-year survival of PF-ILD compared to a cohort of 257 IPF patients collected in our center

Median FVC was 56% (2.29 L), 56% (2.07 L), 50% (1.95 L), at time points − 24, − 12, − 6 before initiation, 44% (1.59 L) at time of initiation and 46.5% (1.77 L) at 6 months after initiation of antifibrotic treatment. Median FVC difference was significant between − 12 and − 6 months before initiation (*p* = 0.004) and also between − 6 and time of initiation (*p* = 0.005), while no further significant decline was reported between time of initiation and + 6 months (*p* = 0.17). This last result was obtained comparing data of only eight patients as three patients died before this time due to respiratory failure (Fig. 2). Antifibrotic drugs were generally well tolerated. Mean dosage of pirfenidone was 2242 ± 337 mg·day^− 1^, while for the patient treated with nintedanib mean dosage was 300 mg·day^− 1^. A dose reduction was needed in 2 cases under pirfenidone treatment: one for rash (case 3) and the other for nausea (case 9), while an early termination was needed in 3 cases due to death following respiratory failure (Table 2). Only one patient experienced acute exacerbations after antifibrotics initiation. Median survival time was 11.1 months since antifibrotic drug initiation (Fig. 3).

## Discussion

In recent years, undeniable progress has been achieved in understanding the pathogenic mechanisms of IPF. This has progressively led to the advent of pirfenidone and nintedanib, the first two drugs able to reduce lung function decline [12–17]. Comparable to IPF, some PF-ILDs are triggered by repetitive lung parenchymal injuries and demonstrate TGFβ-mediated fibroblast activation and myofibroblast accumulation that may lead to a progressive phenotype [18–21]. However, the reasons by which some ILDs demonstrate a IPF-like behavior while some others do not are still unsolved and can be only partially justified by these simple pathogenic similarities.

Corticosteroids represent current first line therapeutic approach with the addition, in some cases, of immunomodulators. However, evidence has clearly demonstrated that a significant proportion of these patients does not benefit of these therapies [11]. Therefore, there is an emerging need to identify possible effective treatments for these specific setting. Due to their antifibrotic and anti-inflammatory activity, both pirfenidone and nintedanib, are potential therapeutic candidates for the management of PF-ILDs. In this context several trials are currently investigating the use of antifibrotic drugs in other progressive, fibrosing ILDs than IPF [19]. For example, the German RELIEF trial assesses the efficacy and safety of pirfenidone in patients with fibrosing ILDs with a progressive phenotype other than IPF [6]. After an exploratory safety trial with Pirfenidone in patients with Systemic sclerosis associated-ILD (SSc-ILD) (LOTUSS trial) has been published, currently the scleroderma lung trial III assesses the efficacy of pirfenidone on the background of Mycophenolate mofetil [7, 19]. Similarly, Pirfenidone is investigated in unclassifiable ILD and in other subgroups of PF-ILDs [19, 27]. Furthermore, nintedanib is investigated in patients with fibrosing, progressive ILDs in the INBUILD trial and results from the SENSCIS trial investigating nintedanib in SSc-ILD are expected soon [5, 8, 19]. However, reports, especially on real life data on the efficacy and safety of antifibrotic drugs in non-IPF PF-ILDs are still lacking.

**Fig. 4:**
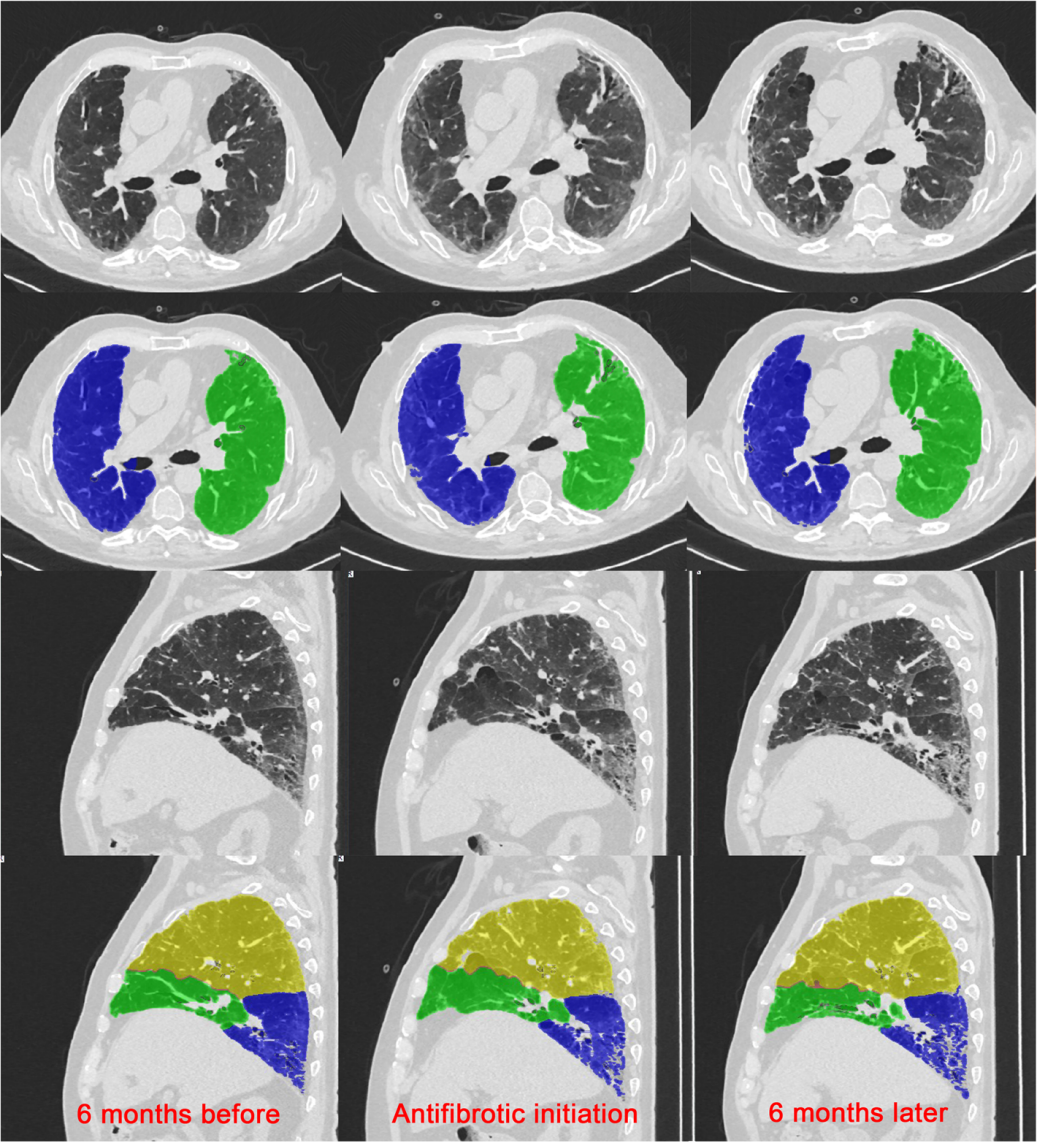
Example of one case showing radiological worsening in the 6 months preceding antifibrotic initiation and no significant changes after 6 months since their initiation. Figure also shows the fully automatic lung parenchyma segmentation as obtained by in-house YACTA software

To our knowledge, our report on the effects of antifibrotic drugs in non-IPF fibrosing and progressive ILDs is the largest and the most heterogeneous reported experience on the use of antifibrotics in this group of PF-ILDs [28–30]. According to our analyses, the introduction of antifibrotics demonstrated a significant reduction of lung function decline (Fig. 2) and of radiologic worsening (Fig. 4) after 6 months since their initiation. Similarly, longitudinal changes in the 40th and 80th percentiles of attenuation histogram, that represent promising radiologic parameters for monitoring the disease extent, demonstrated a progressive increase before antifibrotics initiation and a stabilization after their initiation highlighting a worsening of low density areas (40th percentiles) such as ground-glass areas and of high density areas (80th percentiles) such as fibrotic areas (Fig. 1). These results, even if derived from a limited number of patients, are promising and suggest that antifibrotics may be helpful also in this subset of patients. Interestingly, as some patients may also be classified as IPAF, antifibrotics could represent a valid therapeutic option also for this specific group [22–24]. Furthermore, as documented in IPF patients, both pirfenidone and nintedanib were generally well tolerated, with the need of a dose reduction only in a few cases [12–17].

The one-year survival of PF-ILDs looks incredibly similar to IPF making the two diseases very similar (Fig. 3). However, the pronounced mortality in our cohort has to be also discussed in light of the already very advanced patient status and their impaired general condition. With the exception of one case who experienced a further acute exacerbation after antifibrotic initiation, no other severe adverse events were registered after the introduction of antifibrotics, confirming an acceptable safety and tolerability profile also in PF-ILDs.

This study has some strengths. Patients were evaluated in an academic center through a multidisciplinary discussion, were routinely followed every 3–6 months with pulmonary function tests and visits, and refer to a real-life setting. Moreover, even if the total number of patients is small, there is a variety in the type of PF-ILDs analyzed.

However, there are also several limitations. First of all, this is a retrospective and single-center study. This element might have created some bias of selection and lack of some data. Second, the number of patients is very small. Therefore, results have to be considered with caution and need to be confirmed by clinical trials that are still ongoing. Third, again due to the small number of patients, the statistical approach was very simple and limited to a comparison of median FVC before and after antifibrotics introduction. Finally, due to the observational and retrospective nature of the study, a comparison with an untreated control group was not possible as well as to collect also data on quality of life over the time.

## Conclusions

Antifibrotic treatment after multidisciplinary team discussion and with patient consent may be a valuable treatment option in patients with progressive fibrosing non-IPF ILDs that do not benefit from corticosteroids and immunosuppressive treatments if no other treatment options exist. However, prospective, randomized clinical trials are urgently needed to assess the real impact of antifibrotic therapy in these patients.

## Data Availability

The data analyzed in the current study are not publicly available but may be made available from the corresponding authors on reasonable request.

## Abbreviations

BAL: Bronchoalveolar lavage
DLCO: Diffusing capacity of the lung for carbon monoxide
FVC: Forced vital capacity
HRCT: High resolution computed tomography
IPAF: Interstitial pneumonia with autoimmune features
IPF: Idiopathic Pulmonary Fibrosis
MDT: Multidisciplinary team
PF-ILDs: Progressive fibrosing non-IPF interstitial lung diseases
PPFE: Pleuroparenchymal fibroelastosis
SSc-ILD: Systemic sclerosis associated-ILD
